# Achieving Equity in Generative Artificial Intelligence in Cardiovascular Medicine

**DOI:** 10.1016/j.jacadv.2026.102967

**Published:** 2026-07-04

**Authors:** Aparna Kulkarni, Shyam Visweswaran

**Affiliations:** a Northwell Health, Cohen Children’s Medical Center, Cohen Children’s Heart Center, New Hyde Park, New York, USA; b Department of Biomedical Informatics, Center for Clinical Artificial Intelligence, University of Pittsburgh, Pittsburgh, Pennsylvania, USA

**Keywords:** artificial intelligence, generative AI, health equity

## Abstract

Artificial intelligence (AI), specifically generative AI, presents opportunities to advance equity through transformative practices in cardiovascular care. In this review, we describe how generative AI models risk perpetuating inequities through representation bias, measurement and labeling bias, deployment bias, and contextual issues. We propose a framework for equitable generative AI that addresses the bias types using design principles of representation and contextualization, transparency, accountability, and community engagement supported by operational strategies of digital equity and continuous recalibration. We explore how generative AI has the potential to improve access to specialty care, enhance patient-centered care, support health literacy, and broaden clinical trial participation. Finally, we encourage and propose governance and oversight of generative AI models across all stages of the AI lifecycle.

## Highlights

• Generative AI may worsen cardiovascular disparities in underserved communities.
• A lifecycle equity framework guides bias-aware CVD AI design.
• Equity-centered governance is vital to close cardiovascular disparities.

Cardiovascular disease (CVD) accounts for over 36% mortality worldwide and 50% mortality in the United States.^1^ Non-Hispanic Black, Indigenous, rural, and lower-income individuals have a higher likelihood of hypertension, heart failure, stroke, and reduced access to care and advanced therapies.^1^ These disparities reflect the structural inequities within society, health care systems, living environments, and access to care. The rapid expansion of artificial intelligence (AI) technologies presents opportunities for transformative practices and achieving health equity. These emerging technologies, particularly generative AI, present both an opportunity to address the structural inequities and a risk of deepening them if developed without deliberate attention to equity. Generative AI refers to a type of AI that creates new, human-like content, such as text, images, or audio, based on patterns learned from large data sets. A significant type of generative AI is the large language model (LLM), which specifically focuses on generating human-like text.^2^ Generative AI, including LLMs, can interpret complex medical data, analyze it, and offer recommendations for clinical care. There are numerous applications in CVD aimed at enhancing clinical decision-making, such as automated interpretation of electrocardiograms (ECGs) and echocardiograms, arrhythmia detection, and automated risk prediction scores. Health equity is the state in which everyone has the opportunity to attain full health potential, and no one is disadvantaged from achieving this potential because of social position or any other socially defined circumstance.^3^ In cardiovascular care, this means ensuring equitable access to prevention, early detection, and guideline-directed therapies across race, ethnicity, sex, socioeconomic status, and geography. It is critical that these AI applications be purpose-built not only to uphold this goal but also to advance it.

In this review, we examine the complex relationship between generative AI and health equity in CVD and explore how these technologies can both advance and threaten equitable health care delivery. We discuss the mechanisms underlying model biases, provide recommendations for designing equitable applications, and suggest operational strategies to achieve health equity. Finally, we will outline applicable principles of governance (Central Illustration; Table 1).

**Central Illustration:**
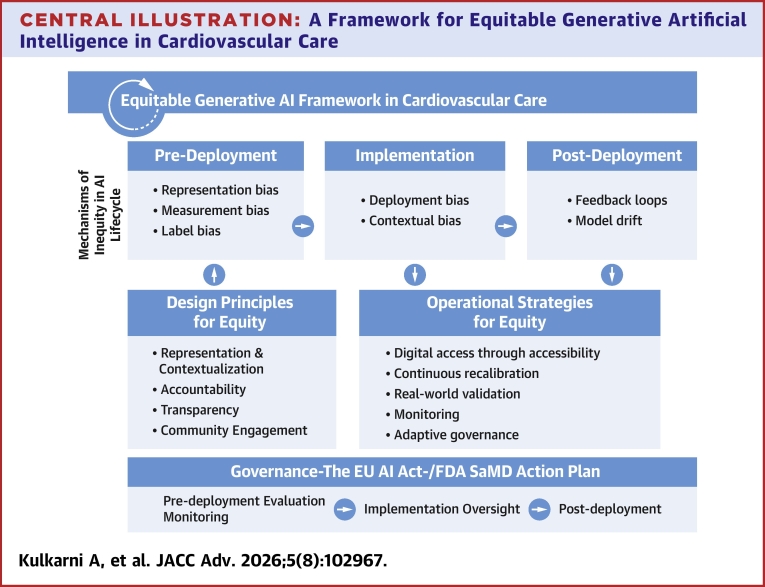
A Framework for Equitable Generative Artificial Intelligence in Cardiovascular Care AI = artificial intelligence; EU = European Union; FDA = U.S. Food and Drug Administration; SaMD = software as a medical device.

**Table 1:** Framework for Equitable Generative AI in Cardiovascular Care: Mapping Bias Types to Design Principles and Operational Strategies

Bias Type	CVD Example	Bias Source	Design Principle	Operational Strategy	CVD-Specific Application
Representation bias	Underrepresentation of Black patients in arrhythmia detection algorithms	Data curation	Representation and contextualization	Digital equity through accessibility	Intentional inclusion of racial/ethnic minorities, women, and rural populations in CVD training data sets; telemedicine to capture underserved populations
Measurement and labeling bias	Pulse oximetry inaccuracy by skin tone; health care cost used as a proxy for CVD need favoring White patients	Data curation	Transparency	Continuous recalibration	Standardized data cards and model cards documenting EHR data provenance; recalibration of CVD risk scores when proxy outcomes misclassify groups
Deployment bias and contextual issues	Academic center CVD models failing in rural or resource-limited settings; LLMs amplifying race-based stereotypes	Data curation and model architecture	Accountability	Continuous recalibration	Local validation before deploying CVD decision-support tools in rural settings; predefined fairness thresholds (demographic parity, equalized odds) triggering recalibration
All bias types	Mistrust reducing data participation from high-CVD-burden communities	Both	Community engagement	Community engagement	Codesign with Black, Indigenous, rural, and low-income communities across the AI lifecycle; community advisory boards; CBPR models such as FAITH!
Model architecture bias	ECG deep learning optimized for aggregate performance is underperforming in minority subgroups despite balanced data	Model architecture	Accountability	Continuous recalibration	Fairness-aware training objectives; subgroup-specific benchmarking; algorithmic auditing across demographic groups

AI = artificial intelligence; CBPR = Community based participatory research; CVD = cardiovascular disease; ECG = electrocardiogram; EHR = electronic health record; LLMs = large language models.

## Mechanisms leading to inequity in generative AI

The lifecycle of generative AI (as well as AI in general) consists of several stages: data creation and processing, model development and validation, model deployment and integration into intended settings, and post-deployment monitoring. Biases that can contribute to health care inequity may arise at any stage of this lifecycle. Below, we briefly describe a few of these biases.^4^^,^^5^

### Representation bias

Representation bias occurs when the data used to train AI do not adequately reflect the target population, resulting in uneven performance when deployed at scale. This bias can stem from selection bias, where only a portion of the target population is sampled, or when the target population differs significantly from the population used during training.^6^

Structural determinants of equity contribute to representation bias. For instance, minority racial groups and women are often underrepresented in legacy medical data sets.^5^ A specific example of this racial underrepresentation is a study by Manrai et al, which found that individuals of African ancestry were undersampled in the genomic reference database. This led to the misclassification of benign variants as pathogenic genes associated with cardiomyopathy.^7^ Furthermore, individuals from certain communities, as well as women, have frequently been underrepresented in clinical trials and databases, which exacerbates the issue of misclassification.^8^^,^^9^ AI trained on biased data can lead to systematic errors. For example, high error rates were observed in Black and Hispanic women for an AI screening tool for chest radiographs.^10^ ECG-based deep learning models have also demonstrated well-documented performance disparities across racial and sex subgroups. A study of 56 automated arrhythmia detection algorithms found a 39% accuracy difference between Black and Asian subjects despite similar aggregate metrics.^11^ Sex-based disparities are equally concerning, with false negative rates significantly higher for female patients, systematically underdiagnosing cardiac disease in women.^11^^,^^12^ Deep learning models can detect race, sex, and age directly from ECG waveforms, raising concerns that models exploit demographic features as shortcuts rather than learning clinically relevant patterns. This has direct implications for equitable CVD diagnosis.^13^ Additionally, LLMs have been found to perpetuate race-based inequities due to their training data improperly representing demographic diversity.^14^^,^^15^

### Measurement and labeling bias

Measurement bias occurs when data are recorded improperly, inadequately, or ambiguously. This issue is especially relevant for electronic health records (EHRs) and medical administrative data, which are collected under uncontrolled conditions^16^ and are increasingly used to develop AI models. For instance, EHRs may contain missing or incorrect information about the race and ethnicity of patients.^17^ Measurement errors can occur in EHR data obtained from medical devices, such as pulse oximeters. For instance, variations in pulse oximetry accuracy across racial groups, due to differences in skin characteristics, have led to systematic racial bias in oxygen saturation readings.^18^

Labeling bias, a type of measurement bias in outcomes, occurs when the labels or outcomes assigned to data reflect human error or the use of inadequate proxies. For instance, diagnostic labels recorded in clinical data may be influenced by clinician judgment, which can be shaped by structural inequities, sociocultural context, or resource limitations, rather than adhering to standardized diagnostic criteria.^16^ This situation can lead to labeling bias, in which differences in diagnostic classifications across populations stem from contextual or systemic factors rather than actual differences in disease prevalence or severity. Proxy outcome measures might capture patterns of health care access rather than the underlying burden of disease, further introducing label bias.^4^ For example, a study revealed that using health care costs as a proxy for health care needs resulted in an AI model that favored affluent White patients over Black patients, despite both groups having similar disease burdens.^19^ Another example of a poor proxy is hospitalization rates, which are often used as surrogate outcomes; populations facing barriers to care, such as financial constraints, limited availability of services, or differing patterns of health care utilization, may be systematically misclassified as having lower risk or disease severity.

### Deployment bias and contextual issues

Deployment bias occurs when an AI model is used in a context different from the one for which it was originally designed. Models trained on data from one setting may demonstrate degraded performance or lack external validity when deployed in a different setting. For example, models validated in high-resource academic centers may not generalize effectively to rural or resource-limited populations without recalibration.^20^ Variations in clinical workflows, patient demographics, and disease prevalence can further limit model generalizability.

Context strongly influences how bias manifests. AI models do not operate in isolation; they interact with social, legal, and institutional structures. Social and historical biases embedded in data may be amplified by AI models,^21^ for instance, reinforcing race-based stereotypes in LLMs.^15^ Some communities have faced long-standing marginalization, leading to health disparities such as obesity, dietary habits, access to health care, and environmental exposures.^1^^,^^22^ When models incorporate data of such communities without appropriate contextualization or adjustment, they risk perpetuating those inequities.

The bias types are not mutually exclusive and may frequently interact across the AI lifecycle. For instance, representation bias from the underrepresentation of patients with darker skin tones used in the training data of pulse oximetry can compound measurement bias, as these AI models may propagate and amplify existing device-level inaccuracies rather than detect them. Recognizing these interdependencies is essential, as interventions targeting a single bias type in isolation may be insufficient to achieve equity.

It is equally important to distinguish between biases arising from curation and those arising from model architecture, as each requires distinct mitigation strategies. Data curation biases, including representation, measurement, and labeling bias, originate in how training data are collected, processed, and annotated prior to model development. These are addressed primarily through intentional data diversification, rigorous data profiling, and standardized documentation practices. Model architecture biases, on the other hand, emerge from design choices made during training, such as setting objective functions, optimizing targets, or using performance metrics that may implicitly prioritize majority-group performance. For example, a generative AI model optimized to aggregate loss across the training population may systematically underperform for minority subgroups whose patterns are underrepresented, even when the underlying data are relatively balanced, as in the ECG model above.^11^ Addressing architecture-level bias requires fairness-aware training objectives, subgroup-specific performance benchmarking, and algorithmic auditing (discussed later), strategies that operate independently of data composition and must be pursued along with data curation improvements. The study by Alday et al retrained their model using an additional constraint, which then demonstrated minimized differences in performance across sex, race, and age. This resulted in a modest reduction in performance, with a significant reduction in bias.^11^

## Design principles for equitable generative AI

Achieving equity in generative AI requires adhering to a set of principles that guide both design and real-world implementation across the AI lifecycle. Equity is not achieved through a single technical decision, but through continuous attention to how data are collected, models are developed and evaluated, and performance is monitored over time. Below, we briefly describe a few design principles for equitable AI-enabled care.

### Representation and contextualization

The development of equitable AI models needs data that reflect the diversity of the populations they serve. Representation should encompass demographic diversity, geographic variation, socioeconomic conditions, and clinical complexity. Data collection strategies should intentionally include underrepresented populations and ensure sample sizes sufficient to support reliable performance across groups. When using existing data, including EHR and administrative data, careful profiling is necessary to identify areas of underrepresentation and misrepresentation.

Since clinical decision-making occurs within complex social and environmental contexts, AI models intended for health care should account for relevant contextual factors, including social determinants of health, access to care, and community-level resources. Particular care is needed in how models represent structural determinants of inequity, since disparities in outcomes often reflect racism, underinvestment, and other systemic conditions rather than inherent differences between groups. Recent National Academy of Medicine publications have therefore argued for approaches that move beyond race-based heuristics and instead attend to the structural conditions that produce unequal health outcomes.^5^^,^^23^ This perspective aligns with the Council of Medical Specialty Societies’ Encoding Equity initiative, which seeks to identify inappropriate uses of race in models and guidelines and to support the redesign of more accurate and equitable decision-making tools.^24^ At the same time, new evaluation frameworks are being developed for generative AI: EquityGuard, for instance, was introduced to detect and mitigate health inequities in LLM outputs through contrastive assessment across clinical scenarios, thereby offering a method for identifying and measuring disparities before deployment.^25^

### Transparency

Transparency is a foundational requirement for establishing trust, accountability, and safe use of AI in health care. Transparent reporting of data provenance, model development processes, intended use, performance characteristics, and known limitations enables users to make informed decisions about adoption and oversight. Insufficient transparency can impede clinical translation and obscure sources of bias or error, thereby undermining patient safety.^26^

Operationalizing transparency requires structured documentation practices across the AI lifecycle. Standardized documentation, such as data cards,^27^ model cards,^28^ evaluation reports, and implementation guides, provides concise, accessible summaries of how data sets were created, how models were trained and validated, and under what conditions they are appropriate for use. National and international guidance emphasizes the need for verifiable, standards-based reporting and post-deployment surveillance to ensure that AI models remain safe, effective, and equitable over time.^29^

### Accountability

Accountability is imperative for ensuring equitable AI models. It requires clearly defined mechanisms for monitoring performance, identifying disparities, and implementing corrective actions throughout the AI lifecycle. This includes regular audits of model performance across different demographic groups and clinically relevant subgroups, transparent reporting of results, and clearly assigned responsibilities for addressing any identified inequities. Of note that evaluating models solely on fairness metrics is insufficient to tackle structural inequities.^30^ Instead, accountability frameworks should include continuous evaluation of clinical impact, which involves comparing predicted outcomes with actual outcomes through audits and ensuring human oversight (mandated European Union [EU] AI Act; recommended in U.S. Food and Drug Administration (FDA) guidance, not mandated).^31^ Accountability for post-deployment surveillance should be explicitly assigned rather than assumed.^32^ At the institutional level, this may be operationalized via cross-functional AI governance committees (details in governance below).^33^

### Community engagement

The biases in AI models do not operate in isolation from the communities they affect. Representation, measurement, and deployment biases have real-world consequences related to the erosion of trust among Black, Indigenous, and other marginalized communities who bear the greatest burden of CVD. Well-documented historical harms from the Tuskegee Syphilis Study to the unconsented use of Henrietta Lacks’ cells and, more recently, from the COVID-19 pandemic have produced enduring and justified mistrust of medical research among these communities.^34^^,^^35^ This mistrust directly shapes data pipelines: communities least willing to participate in data collection due to privacy concerns or prior exploitation are precisely those already underrepresented in CV data sets, thereby compounding representation bias. Privacy concerns in AI applications amplify this dynamic. A survey of racial and ethnic minority patients found that 64% were comfortable with their medical data being used to train AI tools.^36^ Concerns about unauthorized secondary use and loss of control over personal health information are among the most frequently cited barriers to AI acceptance.^37^ In CVD, where community trust is essential for capturing longitudinal data across high-burden populations, these concerns can systematically exclude communities most in need from both the development and adoption of AI tools, perpetuating the very disparities the technology aims to address. Addressing these concerns requires transparent governance, meaningful consent frameworks, and community-based participatory approaches that include marginalized populations, such as Black, Indigenous, rural, and low-income communities that are disproportionately affected by CVD, or mechanisms such as community advisory boards, codesign workshops, and participatory research councils.

Community engagement is essential for developing AI models that reflect the needs and values of the populations they serve. Community members can provide insights into barriers to care, cultural preferences, and local health priorities. Codesign approaches involve patients and community organizations in every stage of the AI lifecycle, including problem definition, data collection, model evaluation, and implementation planning. Such partnerships promote trust, improve adoption, and enhance the relevance of AI models in real-world settings.^38^

The FAITH! (Fostering African-American Improvement in Total Health) Program is one such example of community-based participatory research in CVD. This initiative partnered with Black churches in Minnesota to cocreate a mobile health intervention supported by a digital health advocate network to address multiple CVD risk factors and resulted in sustainable church-based health ministries, increased research participation, and improved CV health and digital health literacy.^39^

## Operational strategies for achieving equity

While design principles provide conceptual guidance, operational strategies translate those principles into practice. These strategies represent actionable interventions that health systems and developers can implement to reduce disparities in AI-enabled care. Without specific attention to these strategies, AI applications could paradoxically widen the digital divide. The following operational strategies are presented in order of increasing implementation complexity. Digital equity through accessibility and continuous recalibration represent strategies that are actionable within existing institutional frameworks, requiring investment in infrastructure and governance processes that health systems can initiate independently.^40^ Community engagement, on the other hand, requires sustained systemic and policy-level commitment to fundamentally restructure the relationship between AI developers, health systems, and the communities most affected by cardiovascular disparities.^39^

### Digital equity through accessibility

Digital access is a critical component of equity, as it significantly influences health outcomes. Over 40% of patients face at least one digital barrier, including limited broadband access, a lack of digital devices, or insufficient digital literacy.^41^ Digital exclusion often overlaps geographically with areas that have a high burden of CVD.^42^ Health systems that rely on digital engagement may unintentionally enhance care for digitally connected populations while marginalizing those without digital access. As a result, the digital divide can worsen the distribution of AI benefits. Generative AI has the potential to help bridge the digital divide by facilitating telemedicine and remote monitoring programs that extend access to expert CVD care to rural and underserved communities.^33^^,^^43^

Language is another crucial aspect of digital equity. AI-based translation tools have shown significant inaccuracies in clinical settings, which can have serious safety and equity implications. These tools must be carefully validated to ensure both accuracy and cultural appropriateness in clinical communication. Ortega et al proposed the Translation, Education, Concordant Care, Community Outreach, Interpretation framework to guide the development of linguistically equitable AI solutions.^44^

### Continuous recalibration based on new evidence

AI models, particularly generative ones, can experience a decline in performance over time as their usage context evolves. To maintain their effectiveness, these models need to be periodically recalibrated to reflect changes in the population and advances in medical practices. One example of such an initiative is the American Heart Association’s Predicting Risk of Cardiovascular Disease Events cardiovascular risk equation. This equation is designed to be sex-specific yet race-free, incorporating neighborhood zip codes alongside CVD risk factors to evaluate long-term risks of atherosclerotic CVD and heart failure and represents an advance over prior risk models.^45^

The Predicting Risk of Cardiovascular Disease Events equation also highlights the challenges in achieving health equity. Although the data used to develop the score are substantial, encompassing 6.6 million adults from 46 cohorts, there are indications that some misrepresentation may still exist. Predictions using this equation indicate that Black adults experience disproportionately higher rates of losing eligibility for statin and antihypertensive therapies compared to White adults. Additionally, men are twice as likely to be affected as women.^46^ These findings emphasize that simply removing race from an equation does not eradicate racial disparities. They also underscore the importance of recalibrating models for their effective application.

Approximately 65% of hospitals in the United States utilize predictive models in health care delivery, and an even higher percentage (79%) rely on models developed from EHR data. However, only 44% of these hospitals have assessed their models for bias.^40^ It is essential for hospitals to conduct local validation before deployment to ensure that the models perform optimally on the patient populations they serve. Models that are validated solely on aggregate data without proper representation may lead to disparities impacting racial and ethnic minorities, women, and socioeconomically disadvantaged groups.^38^

Health systems should establish predefined performance thresholds across demographic subgroups prior to deployment to operationalize recalibration. Recommended fairness metrics include demographic parity (such that positive prediction rates are equivalent across groups) and equalized odds, which require that both true positive and false positive rates are consistent across subgroups.^19^^,^^47^

## The promise of generative AI in enhancing equity in CVD care

Generative AI presents significant opportunities to promote health equity in CVD care, provided they are developed and implemented with a deliberate focus on equity.^43^

### Access to specialty care

As the burden of CVD increases, access to specialty care will become more limited. AI can enhance access to health care by aligning patient demand with available clinical resources. Examples of these systems include virtual front door platforms and intelligent triage solutions that direct patients to the appropriate care pathways.^48^ They can reduce wait times and improve service coordination. When designed responsibly, these systems can minimize variability in care delivery and expand access to specialty expertise. However, these benefits are contingent on foundational infrastructure prerequisites such as reliable broadband connectivity, compatible EHR systems, and adequately trained clinical staff, which are frequently absent in rural and resource-limited settings.

AI can identify and address gaps in preventive care by analyzing EHR data to flag patients who have not received guideline-based screenings or interventions. AI tools may also improve access to care by facilitating telemedicine and remote monitoring programs, extending CVD expertise to rural and underserved areas.^33^^,^^49^^,^^50^ Generative AI, particularly through LLMs, has the potential to democratize access to specialist CVD expertise by providing high-quality clinical decision support services in resource-limited settings.^51^^,^^52^ Furthermore, integrating wearable sensors offers opportunities for continuous CVD monitoring and early detection in populations with limited access to care.^53^ This integration, however, faces substantial infrastructure prerequisites of reliable internet connectivity, device affordability, digital literacy support, and ongoing technical maintenance. Without deliberate investment in these prerequisites, wearable technology risks benefiting only digitally connected populations.

### Patient-centered care

Ambient scribes can help reduce physician burnout caused by extensive EHR documentation, allowing clinicians to spend more time on patient-centered care, particularly for populations with social and language needs.^54^ The equity benefits of these tools depend on validation across diverse patient populations and languages and on accounting for varying levels of digital literacy among patients and clinicians in underserved communities. AI tools can also streamline administrative tasks such as prior authorizations and revenue management, freeing up more time for patient care.^55^

### Patient education and health literacy

Generative AI models can create culturally tailored educational materials in multiple languages and reading levels.^56^ These tools can support shared decision-making and empower patients to manage chronic conditions. Improved health literacy is particularly important for populations with limited access to traditional health education resources.

### Clinical trial recruitment

Generative AI has the potential to improve recruitment of diverse participants into clinical trials by automatically matching eligibility criteria to EHR data^57^ and by supporting more accessible, informed-consent processes tailored to varying levels of health literacy.^58^ Automated screening of clinical data can reduce logistical barriers to enrollment and facilitate the identification of patients from historically underrepresented populations. Increasing participation from diverse populations enhances the external validity and generalizability of clinical research findings and helps ensure that new therapies are safe and effective across the populations they are intended to serve.

### Governance to promote equitable AI

Governance is key to maintaining equity over time. Without effective governance structures, even well-designed AI models may drift toward inequitable performance as populations and clinical practices change. Governance and policy frameworks should address inequity and new regulations, and new frameworks will be necessary to implement them. Creating a public reporting platform through regulatory bodies will enhance accountability for AI tools. Additionally, reimbursement models that incentivize equity-based designs will improve model fairness and the quality of assessments within these frameworks. It is essential to establish cross-functional AI governance committees in health care systems that include representatives from all stakeholders, such as clinical leadership, information technology, compliance, patient safety, privacy, frontline clinicians, and the community.^32^^,^^38^

The Joint Commission and Coalition for Health AI released guidance requiring risk and assessment pre- and post-deployment of AI models, with the publication of vendor-supplied validation data.^32^ They also recommend model cards (mandated in the EU AI Act; recommended by the FDA, not required) that document data characteristics, performance metrics, and known limitations of each AI model. Voluntary safety event reporting to independent entities is suggested. Clinicians and staff should be trained in each AI model based on their roles. The American Heart Association's (AHA’s) science advisory makes similar recommendations and urges the use of the guiding principles of strategic alignment, ethical evaluation, effectiveness assessment, and financial performance in all AI models.^59^

Regulatory frameworks are evolving to address the governance of AI in health care, with the EU and United States each developing distinct but complementary approaches that have direct implications for equitable CV AI deployment. The EU AI Act, entered into force in August 2024,^60^ represents the most comprehensive binding regulatory framework for AI globally and has direct implications for CV AI governance. Most AI systems used in CV clinical decision-making, including ECG interpretation, imaging analysis, and risk stratification, would qualify as high-risk AI systems under the Act, triggering requirements for transparent technical documentation, data governance practices, ensuring demographic representativeness in training data sets, human oversight mechanisms, and mandatory post-market surveillance. Article 10 of the Act explicitly requires that training data be sufficiently representative of the intended use population, one of the few binding legal mandates for addressing representation bias in AI development. The provisions of the EU AI Act are increasingly shaping global standards as multinational developers align their practices with its requirements, despite the Act being directly applicable only to European markets.

In the United States, the Food and Drug Administration regulates AI/machine learning (ML)-based CV decision-support tools as software as a medical device when they meet the device classification criteria.^61^ The FDA's 2021 Action Plan for AI-ML-based software as a medical device established guiding principles, including transparency, real-world performance monitoring, and good ML practices. Its Total Product Lifecycle approach emphasizes continued oversight beyond market authorization. The Predetermined Change Control framework, formalized in 2024 guidance, allows developers to prospectively define anticipated model modifications, including recalibration protocols and associated validation procedures, supporting iterative model improvement without full market resubmission. However, current FDA guidance lacks binding requirements for demographic subgroup performance reporting or standardized fairness metrics as conditions of market authorization, creating a significant regulatory gap compared with the EU AI Act’s explicit data representativeness mandates.

Together, these frameworks represent meaningful progress but share critical gaps; neither mandates specific fairness metrics nor predefined demographic subgroups' performance thresholds as conditions of market authorization. Post-market surveillance obligations lack a standardized criterion for recalibration. Patient notification requirements remain voluntary in the United States and inconsistently implemented in the EU. Closing these gaps will require coordinated international regulatory alignment and enforceable standards for subgroup performance reporting.

#### Pre-deployment evaluation

Before implementation, AI models should undergo rigorous validation to assess performance across demographic groups and clinical settings. Risk assessments should evaluate potential harms, including disparities in outcomes.

#### Implementation oversight

During deployment, health systems should monitor model performance in real time and establish processes for responding to emerging disparities. Training programs should ensure that clinicians understand how to use AI models safely and effectively and report unexpected model behavior. AI vendors should be contractually required to supply updated validation data and disclose known performance limitations across demographic subgroups as a condition of deployment.^59^

#### Post-deployment monitoring

Processes should be implemented for prospective monitoring of data drift in deployed AI models, with integrated automated alert systems triggered when performance falls below predetermined thresholds for demographic subgroups. Such vigilance should enable continuous learning in response to data drift and performance degradation.^62^ The models should be able to automatically update when significant shifts occur.^63^ Automated alert systems can notify clinicians and administrators when performance falls below predetermined thresholds. Vendors retain responsibility for disclosing data drift and providing recalibration support (post-market surveillance mandated in the EU AI Act; monitoring encouraged by the FDA). Regulatory bodies should require public reporting of subgroup performance metrics as a condition of continued deployment authorization for high-risk cardiovascular applications such as arrhythmia detection and risk stratification.^32^

### Cost considerations

Implementing equitable generative AI in CV care carries cost considerations that health systems must anticipate, though the evidence on implementation costs remains nascent. Importantly, these costs are unlikely to be uniformly distributed—under-resourced health systems serving the highest burden CVD populations may be the least equipped to absorb them, reinforcing the need for reimbursement models that incentivize equity-centered design and public-private partnerships that prevent cost barriers from becoming an additional axis of CV AI inequity.

## Conclusions

Generative AI technologies and predictive models stand at a critical juncture in CVD medicine. They have the potential to improve diagnosis, treatment, prevention, and access to care in underserved populations. At the same time, generative AI models are not neutral tools. They reflect the social and institutional contexts in which they are developed and deployed. Achieving equity in generative AI requires a lifecycle approach that integrates diverse data, transparent design, inclusive implementation, and sustained governance. Equity is not a property of models alone. It is a property of systems. By aligning technological innovation with ethical responsibility and community engagement, generative AI can become a powerful tool for reducing CVD disparities and advancing health equity.

Organizations should recognize that achieving health equity requires more than technical solutions; structural changes that address the social determinants of health that produce biased data are also needed. Investment in infrastructure to ensure equitable access, establishment of governance standards, and creating cultures of transparency and accountability are key in achieving these goals.^64^, ^65^, ^66^

## Funding support and author disclosures

Dr Kulkarni has received support from the National Academy of Medicine Scholarship in Diagnostic Excellence. Dr Visweswaran has reported that he has no relationships relevant to the contents of this paper to disclose.
